# Value of information: interim analysis of a randomized, controlled trial of goal-directed hemodynamic treatment for aged patients

**DOI:** 10.1186/1745-6215-14-205

**Published:** 2013-07-09

**Authors:** Erzsebet Bartha, Thomas Davidson, Thor-Henrik Brodtkorb, Per Carlsson, Sigridur Kalman

**Affiliations:** 1 Karolinska Institute, CLINTEC, Division of Anaesthesiology and Department of Anaesthesia and Intensive Medicine, Karolinska University Hospital, Stockholm, Sweden; 2 Department of Anaesthesiology, B 31 Karolinska University Hospital, Huddinge, 141 86, Stockholm, Sweden; 3 Center for Medical Technology Assessment, Linköping University, Linköping, Sweden; 4 RTI Health Solutions, Lund, Sweden

**Keywords:** Expected value of perfect information

## Abstract

**Background:**

A randomized, controlled trial, intended to include 460 patients, is currently studying peroperative goal-directed hemodynamic treatment (GDHT) of aged hip-fracture patients. Interim efficacy analysis performed on the first 100 patients was statistically uncertain; thus, the trial is continuing in accordance with the trial protocol. This raised the present investigation’s main question: Is it reasonable to continue to fund the trial to decrease uncertainty? To answer this question, a previously developed probabilistic cost-effectiveness model was used. That model depicts (1) a choice between routine fluid treatment and GDHT, given uncertainty of current evidence and (2) the monetary value of further data collection to decrease uncertainty. This monetary value, that is, the expected value of perfect information (EVPI), could be used to compare future research costs. Thus, the primary aim of the present investigation was to analyze EVPI of an ongoing trial with interim efficacy observed.

**Methods:**

A previously developed probabilistic decision analytic cost-effectiveness model was employed to compare the routine fluid treatment to GDHT. Results from the interim analysis, published trials, the meta-analysis, and the registry data were used as model inputs. EVPI was predicted using (1) combined uncertainty of model inputs; (2) threshold value of society’s willingness to pay for one, quality-adjusted life-year; and (3) estimated number of future patients exposed to choice between GDHT and routine fluid treatment during the expected lifetime of GDHT.

**Results:**

If a decision to use GDHT were based on cost-effectiveness, then the decision would have a substantial degree of uncertainty. Assuming a 5-year lifetime of GDHT in clinical practice, the number of patients who would be subject to future decisions was 30,400. EVPI per patient would be €204 at a €20,000 threshold value of society’s willingness to pay for one quality-adjusted life-year. Given a future population of 30,400 individuals, total EVPI would be €6.19 million.

**Conclusions:**

If future trial costs are below EVPI, further data collection is potentially cost-effective. When applying a cost-effectiveness model, statements such as ‘further research is needed’ are replaced with ‘further research is cost-effective and ‘further funding of a trial is justified’.

**Trial registration:**

ClinicalTrials.gov NCT01141894

## Background

Before conducting costly clinical trials, researchers may need to estimate trial costs and convince funding bodies that returns on investment are acceptable. Large clinical trial protocols include an interim analysis plan, whose objectives usually cover (1) safety, efficacy, or ethical issues and (2) assessment of expected difficulties with patient enrolment that *could* influence the trial’s costs and the credibility of its results. Interim analyses of efficacy may or may not be conclusive, depending on the detected effect size and the pre-trial sample size estimation. However, interim analysis results may influence investigators; consequently, standards for managing interim results and for implementing various statistical analysis methods have been recommended [1,2]. When the primary outcome’s statistical uncertainty, as revealed by interim analysis, indicates need for further data collection, researchers may ask the question: Is it reasonable to continue to fund the trial to decrease uncertainty? Increasingly, resource allocation in health care considers the principles of cost-effectiveness in different jurisdictions; cost-effectiveness has become a key criterion for decision makers when deciding which health-care interventions should be made available in collectively funded health-care systems [3]. Because the purpose of a clinical trial is to reach a decision [4], the principles of cost-effectiveness and value for money could be, and perhaps should be, applied to decide, after the interim analysis, if a clinical trial should be extended. The question then becomes whether current data from the interim analysis are sufficient or whether data collection should continue to decrease uncertainty regarding treatment choices. Further, when deciding whether or not to introduce a new technology, it is recommended that such a decision be made by considering the cost-effectiveness of the technology, taking into account all existing evidence and whether additional research is itself efficient. Thus, there is sufficient evidence regarding the cost-effectiveness of an intervention when the costs of undertaking additional research are greater than the benefits of reducing uncertainty [3]. Extending this rationale to clinical trials, after interim analysis, a trial should be continued when the costs of undertaking additional research are less than its benefits, in terms of reducing uncertainty. This article describes such an interim analysis of a randomized controlled clinical trial of aged hip-fracture patients (ClinicalTrials.gov NCT01141894) and the estimation of the value of extending the trial on the basis of value of information analysis. The trial’s objective was to compare costs and consequences of routine peroperative fluid treatment with goal-directed hemodynamic treatment (GDHT). The interim analysis during the trial determined that, due to statistical uncertainty, further data collection was required. Thus our present investigation’s primary and secondary aims were to (1) analyze expected value of perfect information (EVPI) when studying GDHT, and (2) provide decision support material when stakeholders must determine if it is reasonable to fund further data collection after the interim analysis.

Our investigation modeled cost-effectiveness and estimated result uncertainty of the clinical trial. Our model depicted (1) the choice between treatment options, given current, best available evidence; and (2) the monetary value of a decision to collect further information, via future clinical research, to decrease uncertainty. Here, monetary value is expressed as a concept called expected value of perfect information (EVPI) [5,6].

## Methods

This section describes (1) the clinical trial’s design and efficacy data extracted from the interim analysis, (2) the decision-analytic model and model inputs used in our analyses, and (3) the EVPI estimation method.

### Design of the clinical trial, interim analysis efficacy data

Our data source was a single-centre, open, randomized (1:1), and controlled, parallel-group, superiority clinical trial. The trial is on-going at a primary teaching hospital, Karolinska University Hospital, Huddinge, Sweden. Eligible patients (aged ≥ 70 years, weight ≥ 40 kg) are those scheduled for hip-fracture surgery during regular operating hours. Patients were enrolled if a signed informed consent was obtained *after* they received information about trial-specific procedures. The local research ethics committee approved the trial (ID: 2008–1240–31), and Sweden’s Medical Products Agency authorised the trial (MPA ID; 151:2009/81083).

#### Trial objectives

*Primary*: to identify the absolute and relative risk of post-operative complications at hospital discharge. *Secondary*: to analyze cost-effectiveness of GDHT compared with routine fluid treatment.

#### Statistics

Using expected absolute risk (0.61) [7] and relative risk (0.63) [8] for post-operative morbidity (GDHT compared with routine fluid treatment), a sample size calculation was made (80% power, the type I error <0.05). Assuming that 57% of complications could be reduced due to patients’ age and comorbidity [9], a 460-patient sample size is required. For the analysis Statistical software was used (version 10. StatSoft Inc, Tulsa, OK, USA).

#### Interventions

Interventions included the following: (1) GDHT to attain oxygen delivery index >600 mL/ min^-1^ m^-2^ (using fluids and dobutamine) and (2) a protocol-guided routine fluid treatment. Both groups were monitored with the LiDCO™ monitor ( LiDCO Ltd., Sawston, Cambridge, United Kingdom).

#### The effect size per interim analysis

**Figure 1:**
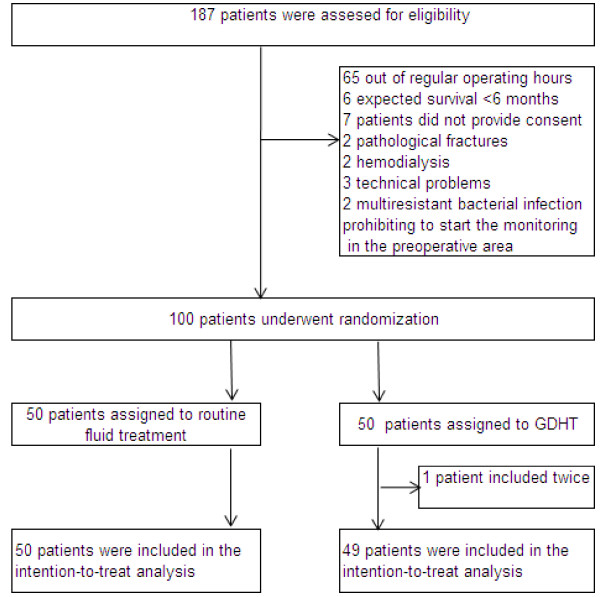
**Inclusion sequence of the first 100 randomized patients in the trial:ClinicalTrials.gov NCT01141894.**

This article reports relative risk of post-operative complications from the trial because these data were specified in the interim analysis strategy. See Figure 1 for the inclusion sequence and Table 1 for patient characteristics. The post-operative outcome is expressed by relative risk (95% confidence intervals) taken from an intention-to-treat analysis. In-hospital mortality (n = 3) contributed to post-operative complications.

**Table 1:** Patient characteristics and interim efficacy data; values are absolute or mean ± SD

**Patient characteristics**	**GDHT**	**Routine fluid treatment**
Number allocated	49	50
Age, years (mean)	86 (± 7)	85 ( ± 7)
Sex, male/female	13/36	9/41
American Society of Anesthesiologists’ grading (1/2/3/4)	0/13/31/5	1/15/29/5
Number of patients with complications (including in-hospital mortality)	15	19
Relative risk of GDHT compared to routine fluid treatment based on intention to treat (95% CI)	0.806 (0.464 to 1.397)	

*CI* confidence interval, *GDHT* goal-directed hemodynamic therapy.

### The model and model inputs used in this investigation

**Figure 2:**
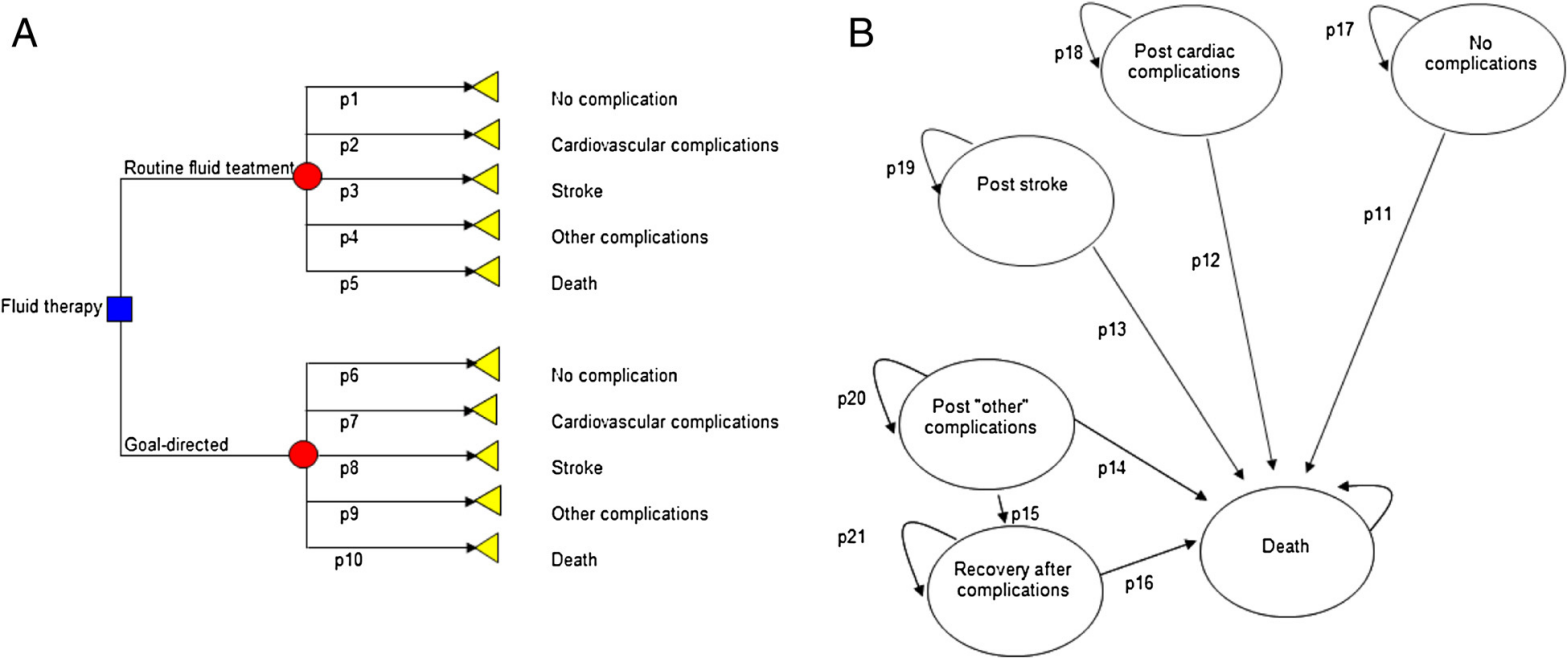
**Cost**-**effectiveness model. A)** Short-term model, the decision tree. The arrows represent the transition of the hypothetical patients towards the selected post-operative outcomes (triangles). These transitions are characterized by probability estimates (*p*1 to *p*10), costs, and health-related quality-of-life weights. For the routine fluid treatment, probability estimates were extracted from a cohort from Lund University Hospital [7]. * For goal-directed hemodynamic treatment (GDHT), the interim analysis was used. ** For mortality, published data on high-risk patients were used [8]. **B)** The long-term model, Markov structure. The hypothetical patients were allocated to health states characterized by health-related quality-of-life weights. During annual cycles of simulation, the patients transition in the model or stay in the same heath state. These transitions are characterized by probability estimates (*p*11 to *p*21). {AU After each cycle, quality-adjusted life-years and direct health-care costs are aggregated.

A decision-analytic probabilistic cost-effectiveness model (Figure 2) was developed. The model illustrates the consequences of use of GDHT or routine fluid treatment for a hypothetical cohort of patients after hip fracture during 5 post-operative years [10]. Model inputs consisted of these estimates: (1) probability of post-operative morbidity and mortality, (2) health-related quality of life, and (3) costs. Model outputs consisted of (1) aggregated health-care costs and (2) quality-adjusted life-years (QALYs). The model was programmed and analyzed using Microsoft Excel (2007, version 12.0.6425.1000). The analysis applied Swedish hospital costs that were converted to euros using the exchange rate of €1 = SEK 9.41. Costs and QALYs were discounted by 3% annually.

More comprehensive descriptions of the model structure and data used for model parameters are available elsewhere [10] (Additional files 1 and 2). The model contained two parts: a decision tree, used to estimate short-term costs and effects, and a Markov structure, used to estimate long-term costs and effectsThe decision-tree in Figure 2A illustrates short-term outcome for hypothetical patients. The tree starts with the decision (rectangle) between the two alternatives, followed by the chance node (circle), whereby the patients can transition (by chance) along the arrows to selected, post-operative outcomes (triangles). These outcomes are as follows: uncomplicated recovery; cardiovascular complications; stroke; other complications (that is, pulmonary or urinary tract infections, post-operative confusion, kidney insufficiency, wound infections, and pulmonary embolism); or death.

#### Model inputs in the decision tree

The model inputs in the decision tree (Table 2) consisted of (1) probability estimates of the post-operative outcome, (2) estimates of the post-operative health state (expressed by QALY weights), and (3) costs.

**Table 2:** Model inputs

**Model inputs**	**Estimates**	**Distributions**
Short-term clinical outcome (routine care)^a^		
Probability of…		
In-hospital mortality	0.129	Dirichlet^I^ (52, 26, 2, 162,160)
Cardiovascular complications	0.065	
Stroke	0.005	
Other complications	0.403	
uncomplicated recovery	0.398	
Relative risk of mortality GDHT versus routine [8]	0.75	Lognormal (−0.28; 0.09)
Relative risk of complications GDHT versus routine	0.81	Lognormal
Long-term clinical outcome (routine care)		
Mortality associated with cardiovascular disease^b^		
First year	0.107	Deterministic
Second year	0.058	Deterministic
Third year	0.056	Deterministic
Mortality associated with stroke at 3 months^c^	0.15	Deterministic
Mortality associated with other complications^a^	0.18	Beta (31; 140)
Recovery associated with other complications^a^	0.41	Beta (70; 101)
Mortality after recovery with other complications^a^	0.15	Beta (17; 95)
Costs/patient for routine fluid treatment in the clinical routine (€)^d^		
Medical device for fluid treatment	11	Deterministic
Human resources in pre-operative area	27	Deterministic
Human resources during anesthesia	117	Deterministic
Costs/patient for GDHT (€)^d^		
Medical device for GDHT	221	Deterministic
Human resources in pre-operative area	159	Deterministic
Human resources during anesthesia	401	Deterministic
Post-operative direct health-care costs/patient (€)^e^		
Cardiovascular complications		
Myocardial infarction	7,498	Gamma (90; 83)
Heart failure	9,903	Gamma (104;95)
Stroke	7,550	Gamma (8; 956)
Other complications		
Pneumonia	8,514	Gamma (106; 81)
Renal failure	12,197	Gamma (6; 1442)
Wound infection	8,566	Gamma (218; 39)
Deep-vein thrombosis	7,617	Gamma (62; 124)
Pulmonary embolism	10,190	Gamma (17, 600)
Gastrointestinal bleeding	9,900	Gamma (64, 154)
Confusion	7,961	Gamma (866; 9)
Death	9,020	Gamma (273, 33)
No complications	6,753	Gamma (956; 7)
Direct health-care costs, first year after hospital stay (€)^f^		
State after…		
No complications	147	Deterministic
Cardiovascular complications	7,673	Deterministic
Stroke	7,512	Deterministic
Other complications	7,314	Deterministic
Recovery from other complications	396	Deterministic
Death	4,837	Deterministic
Direct health-care costs, 2 to 10 year after hospital stay (€)^f^		
State after…		
Cardiovascular complications	386	Deterministic
Stroke	402	Deterministic
Other complications	396	Deterministic
QALY weights, estimates [7]		
>80 years age	0.74	Beta (322; 113)
Recovered after other complication [9]	0.66	Beta (227; 117)
Decrements of QALY weights [8]		
State after…		
Cardiovascular complications	−0.19	Gamma (298; 0.0006)
Stroke	−0.35	Gamma (100; 0.0035)
Other complications	−0.15	Gamma (100; 0.0007)

*GDHT* goal-directed hemodynamic therapy, *QALY* quality-adjusted life years, € Euros.

^I^ The Dirichlet distribution is a multivariate normalization of beta distribution that considers that the sum of probabilities is 1.0.

^a^ Swedish Registry on Hip Fracture.

^b^ Swedish National Registry on Secondary Prevention in Cardiac Intensive Care.

^c^ Swedish National Stroke Registry.

^d^ Karolinska University Hospital in Huddinge, Sweden.

^e^ University Hospital in Lund, Sweden.

^f^ Epidemiological Centre of the Swedish National Board of Health.

Probability estimates characterized each post-operative outcome (the post-operative complications as well as death).

Probability estimates for routine fluid treatment, by type of post-operative complication, could not be calculated from the interim data due to the low number of observations per complication (n <5). Instead, these estimates were extracted from a Swedish patient cohort with hip fracture.[7] For GDHT, the estimate of relative risk from the interim analysis was applied to the probability estimates for the routine fluid treatment by type of post-operative outcome (Table 2). Mortality estimates could not be calculated from the interim data due to the low number of observations (n <5); instead, published meta-analysis data were used [8].

Pre-fracture QALY weights were obtained from an age-matched general population [11] (aged >80 years). Post-fracture QALY weights associated to the selected post-operative outcomes were unavailable, so QALY weights were obtained from a representative sample with the disease in the population [11,12] and from a longitudinal Swedish clinical trial [13].

The long-term model (Markov structure) extrapolated the effects of the post-operative outcome to post-operative health conditions and the influence of the outcome on long-term survival (Figure 2B). Hypothetical patients transitioned among these health conditions or stayed in the current condition during the cycles in 1 year (thatis, a Markov cycle). Recovery is *allowed* from the ‘other complications’ state; we assumed that patients continued to live with cardiovascular complications or stroke after the initial event. During each Markov cycle, quality of life and number of survivors decrease.

#### Model inputs in the Markov structure

Model inputs in the long-term model (Table 2) consisted of survival data, QALY weights, and cost data.

For patients without complication, age-adjusted standard mortality was used. For patients with complications, disease-related mortality was used (non-fractured patients with cardiovascular, cerebrovascular or with other diseases). In instances of cardiovascular complications or stroke, survival was estimated using age- and disease-related mortality from the Swedish National Stroke Registry [14] and the Swedish National Registry on Secondary Prevention in Cardiac Intensive Care (Kalle Spångberg, Ph.D., section manager, Uppsala Clinical Research Center, Uppsala University, Uppsala, Sweden, written communication: 15 May 2009).

QALY weights associated with short-term clinical outcomes were allocated in the early simulation in the Markov structure. The post-fracture QALY weights were multiplied by the time spent in the current state of health, which resulted in the number of QALYs.

Mean costs of inpatient and outpatient long-term medical care services were obtained from the Epidemiological Centre of the Swedish National Board of Health and Welfare for patients undergoing surgery for hip fracture in 2007 and hospitalized during 2008 (Leif Forsberg, statistician, Department of Statistics, Monitoring and Evaluation, Swedish National Board of Health, Stockholm, Sweden, written communication: 7 December 2009). Hospital costs for each post-operative complication were acquired from individual patient-specific cost data obtained from University Hospital in Lund, Sweden.

#### Assumptions in the model

The following assumptions were made in the model:

1. Hypothetical patients could have one post-operative complication.

2. Patients experiencing a complication had a decline of quality of life during the 5 post-operative years equal to that of patients in the non-fractured population who had cardiovascular complications or stroke.

3. The 5-year survival rate after uncomplicated recovery for patients with hip fracture was the same as that for aged non-fractured patients (standard mortality).

4. The 5-year survival rate for post-operative cardiovascular complications and stroke in patients with hip fracture was the same as that in non-fractured patients with cardiovascular complications.

#### Probabilistic sensitivity analysis

To incorporate the uncertainty of model parameters in the probabilistic sensitivity analysis, each parameter associated with statistical uncertainty was defined with probability distributions. The choice of distributions was guided by recent recommendations by Briggs *et al*. [6] and was parameterized with the mean and standard error. The values of QALY weights are constrained on the interval 0 to 1 and for these beta distributions were used. The transition probabilities are mutually exclusive events (multinomial data) and for these Dirichlet distribution was used that is a multivariate generalization of beta distribution. The skewed cost data and the decrements of QALY weights constrained on the interval 0 to positive infinity, and for these parameters gamma distribution was used. The relative risk (ratio) was transformed into a log form and log-normal distributions were used. In Table 2 the distributions used are listed for each parameter. The long-term costs were based on fixed price lists, and the survival rates after cardiovascular complications or stroke were extracted from registries on large populations with low standard error (Table 2). Thus, both these sets of parameters were incorporated as deterministic probabilities in the model.

**Figure 3:**
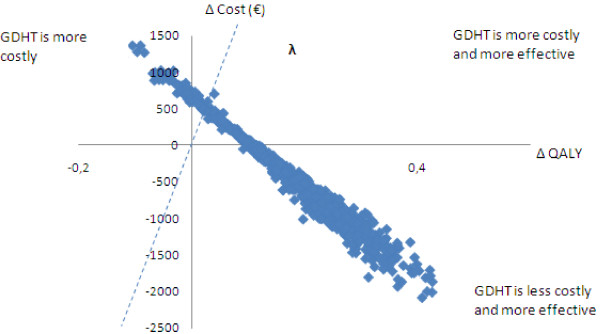
**Incremental costs and effects (∆*QALY*) of goal-directed hemodynamic treatment (GDHT) versus routine fluid therapy.** The dotted line represents one threshold value of how much society would be willing to pay for 1 additional life-year with full health for each patient in the target population.

To propagate the uncertainty of the parameters through to the cost-effectiveness estimate, a second-order Monte Carlo simulation was used; the cohort was simulated through 5 Markov cycles (years). The input parameters were sampled using the distributions given in Table 2. In each simulation, the set of model input values was randomly drawn from the model parameter’s defined probability distributions. The simulation then was performed 1,000 times [6], generating 1,000 estimates of aggregated costs and QALYs. In Figure 3, incremental costs (∆C = Cost_GDHT_ - Cost_Routine_) are plotted against incremental effects (∆E = Effect_GDHT_ - Effect_Routine_). For 91.5% of simulations, GDHT (compared with routine fluid treatment) was better and less costly (∆E positive and ∆C negative) or was better and more costly (both ∆E and ∆C positive).

### Expected value of perfect information estimation method

As shown in Figure 3, the model propagated parameter uncertainty onto the uncertainty of cost-effectiveness. If a decision was to be made regarding adoption of GDHTon the basis of expected costs and QALYs from the current evidence, this decision would be associated with uncertainty and there would be a risk of making an incorrect decision. In Figure 3, the number of values in upper-left quadrant (GDHT more costly and less effective) contributes to the probability of making such a decsion. In addition, some values in the upper-right quadrant also may represent an incorrect decision - despite GDHT resulting in positive incremental effect (∆E) - because the cost per QALY may be perceived as being too high. The primary decision-making factor in this quadrant is a threshold value of how much society is willing to pay for 1 additional life-year with full health in the target population. The slope of the dotted line (λ) on Figure 3 illustrates a value of €20,000 per QALY. The line represents one possible threshold value (or cost-effectiveness threshold); values above the dotted line represent an incorrect decision.

The combination of uncertainty of model inputs and society`s willingness to pay for 1 additional life-year with full health influences the decision. The probabilities of making an incorrect decision are quantified, given the uncertainty of model inputs and the consequences of the relinquished or foregone benefit if GDHT were adopted.

The purpose of further data collection is to eliminate risk of relinquished benefit. In practice, the value of this relinquished benefit is the EVPI, whereby a decision is made for one future patient. The EVPI must be calculated for all potential future clinical decisions on all future patients who will gain by the advantage of additional research during the treatment’s (for example, GDHT) expected lifetime in clinical use. Therefore, the effective population, that is, the population that may benefit in the future during the treatment’s lifetime, is estimated (and the discount rate is applied). The total EVPI population is calculated by multiplying the EVPI per patient by the population that might benefit in the future (effective population):

$$\text{Population}\;\text{EVPI}=\text{EVP}I_{\text{patient}}\times \text{effective}\;\text{Population}$$

## Results

Figure 3 illustrates the model outputs: expected costs and effects. It shows that in 76.2% of the simulations, the GDHT is less costly and more effective (negative incremental cost, positive incremental effect); in 17.1% of simulations, GDHT is more costly and more effective (positive incremental cost and positive incremental effect); and in 6.7% of simulations, GDHT more expensive and less effective (positive incremental cost and negative incremental effect).

### Expected value of perfect information

**Figure 4:**
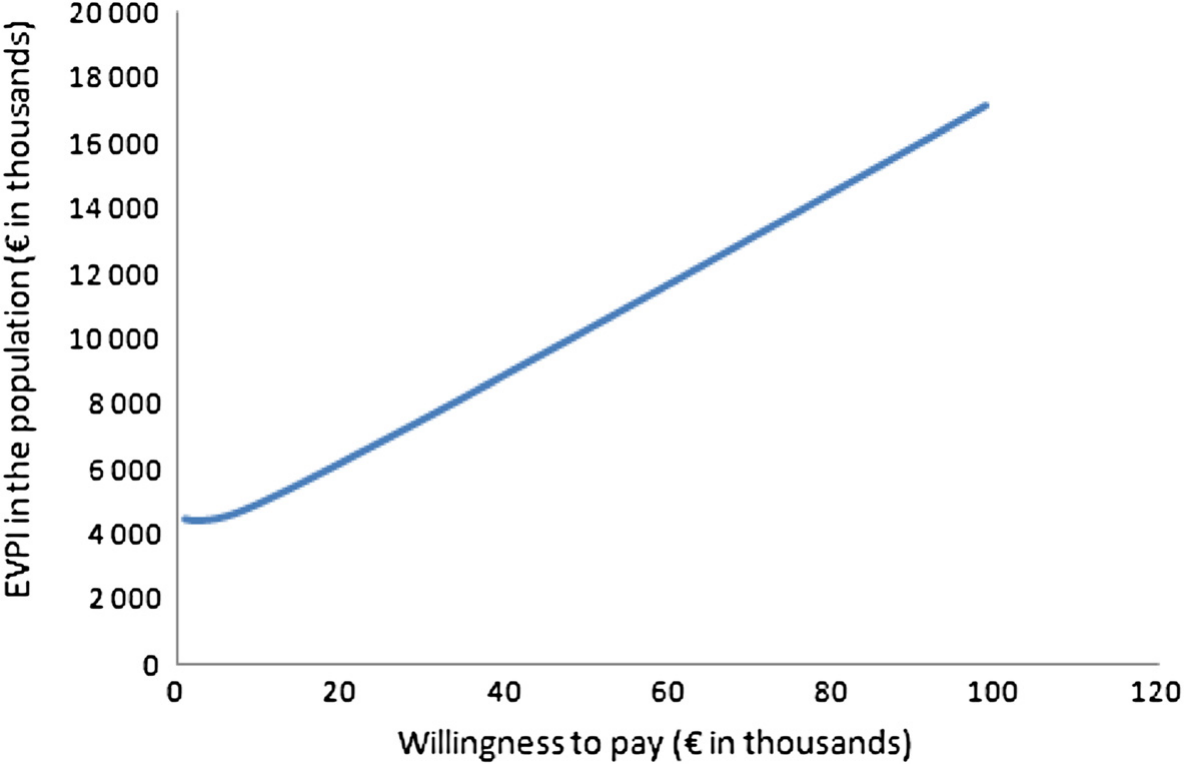
**The expected value of further information for the Swedish patient population aged >80 years with hip fracture.** The expected value of perfect information (EVPI) is plotted against the willingness to pay per quality-adjusted life-year (cost-effectiveness threshold).

At a cost-effectiveness threshold (λ) of €50,000, the EVPI is €337 per patient. At a threshold of €20,000, the EVPI per patient is €204. Given 6,440 operations annually in Sweden (for patients aged >80 years), a conservative expected lifetime of 5 years for the GDHT technology, and a 3% discount per year, the EVPI for the effective population (30,378 individuals) is €6.19 million to €10.2 million when the cost-effectiveness threshold is between €20,000 and €50,000. (The authors assumed that new technologies or other innovations may influence clinical practice and that GDHT may not be the current trend after 5 years.) The model was run using varying values of a cost-effectiveness threshold, which generated varying expected values of perfect information (Figure 4).

## Discussion

After classical statistical inference analysis of the interim efficacy data, our model showed that the GDHT should not be adopted in the clinical routine because the null hypothesis (relative risk = 1.0) was within limits of the 95% confidence interval (Table 1). However, the cost-effectiveness analysis and its accompanying probabilistic sensitivity analysis, taking existing evidence into account, showed that, in 93.3% of the simulations, treating patients with GDHT, when compared to routine fluid treatment, resulted in QALYs gained (Figure 3). However, if a decision was made on the basis of current information, some iteration found that the decision could be incorrect where there are higher health-care costs and less effect (upper-left quadrant on Figure 3) or when costs are above the cost-effectiveness threshold (upper-right quadrant). The EVPI is the value of the incorrect decision as it is the maximum acceptable value that society should pay to eliminate decision uncertainty via further data collection. The cost-effectiveness is a related value that society is willing to pay for 1 additional life-year with full health (cost-effectiveness threshold, λ) for each patient in the population of interest. In Sweden, there is no fixed officially accepted threshold or range, but a cost-effectiveness threshold of between €20,000 and €50,000 has been discussed in this paper; in exceptional cases, this threshold can be even higher. Severity of the health condition under consideration is the major reason for accepting varying thresholds [15-20]. With a broad range of alternative implicit thresholds for society’s willingness to pay for one QALY, we chose a conservative interpretation in drawing our conclusions. For the broad range of cost effectiveness thresholds used in this analysis, we found that the EVPI is in the interval of €6.19 to €10.2 million.

### The rationale of probabilistic modeling

The rationale of probabilistic modeling is to reflect the uncertainty of the model inputs and to describe the uncertainty about the model outputs. In non-linear models, the model outputs are the result of multiplicative or power functions. The interest of a decision-maker is the expected distribution of model outputs. This cannot be obtained by analyzing the upper-limit and lower-limit 95% confidence intervals of the model inputs [21]. Instead, the uncertainty of model outputs is obtained by probabilistic sensitivity analyses [21]. The uncertainty of model outputs implies the possibility of an incorrect decision that results in costs (forgone benefits). In the decision theoretic approach, a value is ascribed to the reduction of uncertainty (creation of additional information) and a decision may include the option to acquire additional information. It is important to note that the current calculated EVPI is conditional on model uncertainty due to the assumptions made (and listed in the methods) about the model structure and the data used. Such assumptions are not accounted for in the distributions of the parameters because they originate from active choices made rather than from statistical uncertainty. The effect of our four assumptions on mean cost-effectiveness and its uncertainty was investigated in the previously published model [10] and was found to have a marginal influence on the results. Thus, our assumptions have not been investigated here with regard to their influence on EVPI.

### The rationale of the expected value of perfect information

EVPI implies that data are obtained from an infinite sample that removes all uncertainty and places an upper limit of the value of further research, given that additional data may reduce uncertainty. In reality, the value of further data collection resulting in new information is most likely far less. Further research may yield partial information on the parameters of interest (not an infinite sample). The value of new information thus depends on the uncertainty of the new parameter estimates. With perfect information, a decision-maker would know how the uncertainties would resolve and could make the correct decision. The better decision would be to choose a treatment with better effect at costs below a threshold value of what the society is willing to pay per gained QALY. EVPI can help decision makers identify which research is potentially cost-effective and which is not. It does not imply that research can be continued until the costs of research are below the EVPI.

Publications regarding the EVPI method for interim analysis appear to be non-existent. In a broader perspective, however, EVPI was recently used for two pilot studies of research priorities in the United Kingdom [22-25]. In our study, we conclude that trial NCT01141894 should be continued, given the uncertain interim efficacy data and the rules of classical statistical inference. However, recruitment time in this on-going trial has been prolonged for logistical reasons (almost 30% of the hip-facture surgeries initially planned during regular operating room hours were performed during off hours (65 out of 187 assessed for eligibility; see Figure 1). This factor increases trial cost; therefore, it is reasonable to address the monetary value of further research because it is not obvious that the trial should be financed for further data collection, given scarce clinical research resources.

### Interim analysis risks

Sharing results of interim analyses could influence an on-going trial, and guidelines request that results from an interim analysis not be made public [1,2,26]. This is important because further recruitment could be affected and because patients could prefer any of the treatments, as seen in the preliminary analysis of efficacy and adverse events (although the intervention is not yet scientifically proven). In addition, the risk of introducing bias into the on-going trial could be substantial [26]. In line with the current recommendations, an interim analysis and the subsequent decision on further data collection should be performed by confidential data safety and monitoring committees. While an EVPI analysis could support such a decision or be used as an argument when applying for further funds, guidelines nevertheless suggest that the results of an interim analysis in such instances not be made public. However, the adherence to these guidelines has been questioned in the past, and the guidelines have been characterized as paternalistic [27]. Researchers in favor of making interim analyses public believe that it is important to inform society, patients, and other researchers and to guide the design of future trials in considering safety issues and sample size. However, it is important to note that current guidelines and statistical methods insist on a blind interim analysis[2,28]. The current trial was randomized but open and blinded during analysis.

## Conclusions

After analysis of interim efficacy data, further data collection in trial NCT01141894 is needed for statistical inference analysis. A further 4 years of recruitment is planned, with a 12-month follow-up period. Even if predicted trial costs increase, it seems reasonable to fund the trial further, provided these costs remain below €6.19 million.

Decisions on funding clinical trials usually do not utilize the results of health economic evaluations of the trials’ interim analyses, even if the trials are very resource intensive and are funded by public resources. With use of the analytic framework, such as cost-effectiveness models, we can replace the statement ‘further research is needed*’* with ‘further research is cost-effective*’* and ‘further funding of a trial is justified.’

## Abbreviations

EQ-5D: EuroQol Group`s questionnaire; EVPI: Expected value of perfect information; GDHT: Goal-directed hemodynamic treatment; QALY: Quality-adjusted life year.

## Competing interests

The authors declare that they have no competing interests.

## Authors’ contributions

EB: main article author, probabilistic cost-effectiveness model development, clinical trial design and launch, interim analysis and EVPI analysis. TD: cost-effectiveness model development and article development. THB: further development of cost-effectiveness model for EVPI analysis and article development. PC: advisor for pre-trial cost-effectiveness analysis and EVPI analysis of interim analysis and article development. SK: advisor for the entire project, probabilistic cost-effectiveness model development, clinical trial design and launch, interim analysis, and article development. All authors read and approved the final manuscript.

## Supplementary Material

Additional file 1 **A decision tree starts with the decision (rectangle) between the two alternatives followed by a circle (a chance node) where alternative events are possible; these are illustrated by branches coming out from the chance node, representing the clinical pathways.** The branches end with triangles representing outcomes. The pathways are mutually exclusive and are characterized by the probabilities; the sum of probabilities following each node is 1.0. Each pathway is associated with health care costs and an outcome (QALY). The expected costs and effects are based on the summation of pathway values weighted by the pathway probabilities. Click here for file

Additional file 2 **The calculated probabilities of survival and mortality following the post-operative complications are illustrated for the routine fluid for five years. Each arrow represents a pathway and the pathways are characterized with probabilities.** Each pathway is associated with costs and quality of life weights. The costs and quality of life weights of each pathway are weighted by the corresponding pathway probabilities. The sum of these weighted costs yields the expected cost during the first year and the weighted quality of life index multiplied by one year yields the expected quality adjusted life year (QALY). The cycles are repeated five times and the aggregated costs and QALYs are calculated. The same calculation is done for the GDHT. Click here for file
