# Supplementary material for: Value of information: interim analysis of a randomized, controlled trial of goal-directed hemodynamic treatment for aged patients
Source: Trials. 2013 Jul 9;14:205. doi: 10.1186/1745-6215-14-205 (PMC3717025; doi:10.1186/1745-6215-14-205)
Supplement: Additional file 1 — A decision tree starts with the decision (rectangle) between the two alternatives followed by a circle (a chance node) where alternative events are possible; these are illustrated by branches coming out from the chance node, representing the clinical pathways. The branches end with triangles representing outcomes. The pathways are mutually exclusive and are characterized by the probabilities; the sum of probabilities following each node is 1.0. Each pathway is associated with health care costs and an outcome (QALY). The expected costs and effects are based on the summation of pathway values weighted by the pathway probabilities. [file 1745-6215-14-205-S1.pdf]

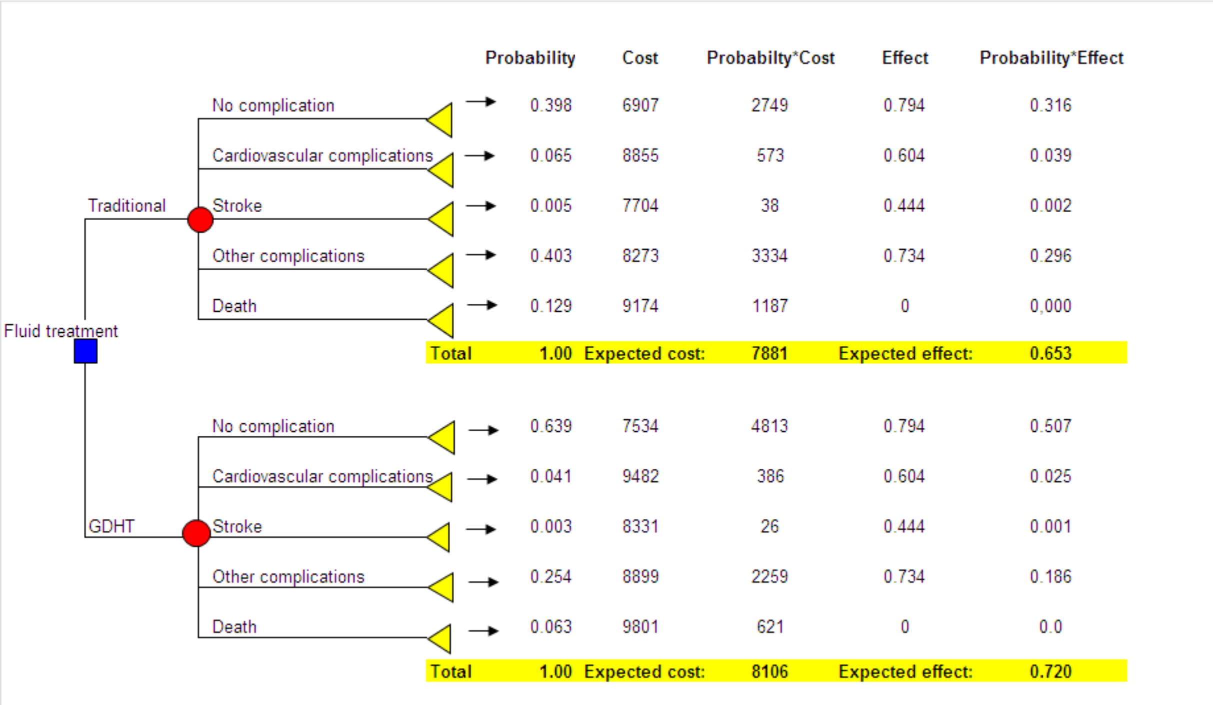

Appendix 1

A decision tree is starts with the decision (rectangle) between the two alternatives followed by a circle (a chance node) where alternative events are possible; these are illustrated by branches coming out from chance node, representing the clinical pathways. The branches are ended by triangles representing outcomes. The pathways are mutually exclusive and are characterized by the probabilities; the sum of probabilities following each node is 1.0. Each pathway is associated with health care costs and an outcome (QALY). The expected costs and effects are based on the summation of pathway values weighted by the pathway probabilities.
