# Supplementary material for: Value of information: interim analysis of a randomized, controlled trial of goal-directed hemodynamic treatment for aged patients
Source: Trials. 2013 Jul 9;14:205. doi: 10.1186/1745-6215-14-205 (PMC3717025; doi:10.1186/1745-6215-14-205)
Supplement: Additional file 2 — The calculated probabilities of survival and mortality following the post-operative complications are illustrated for the routine fluid for five years. Each arrow represents a pathway and the pathways are characterized with probabilities. Each pathway is associated with costs and quality of life weights. The costs and quality of life weights of each pathway are weighted by the corresponding pathway probabilities. The sum of these weighted costs yields the expected cost during the first year and the weighted quality of life index multiplied by one year yields the expected quality adjusted life year (QALY). The cycles are repeated five times and the aggregated costs and QALYs are calculated. The same calculation is done for the GDHT. [file 1745-6215-14-205-S2.pdf]

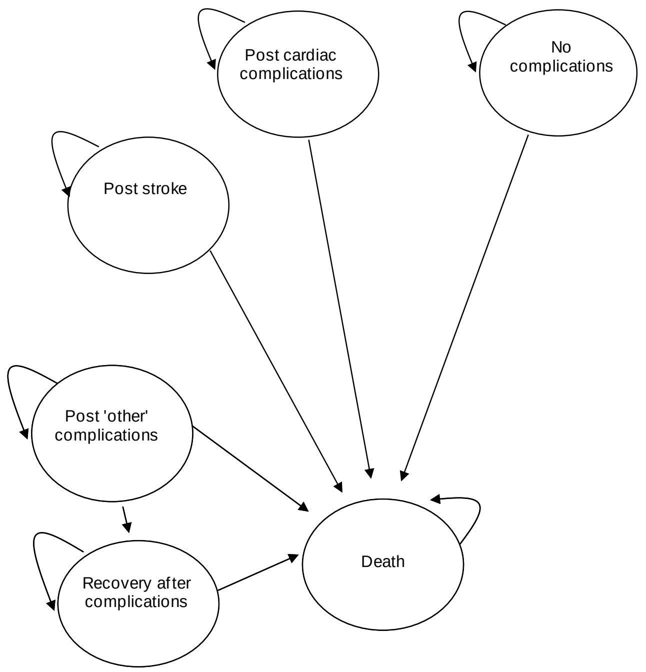

Age	Cycel	Standard mortality	No complication	Cardiovascular complication	Cerebrovascular complication	Other complications	Recovery after other complications	Mortality	Check	Costs	Discounted cost	QALY	Discounted QALY
75.00	0		0.44	0.06	0.0012	0.35	0.00	0.15	1.00	0	0	0.56	0.56
76.00	1	0.06004	0.42	0.05	0.0009	0.12	0.14	0.26	1.00	4346	3140	0.98	0.70
77.00	2	0.065085	0.39	0.05	0.0009	0.06	0.19	0.31	1.00	1067	748	1.69	1.19
78.00	3	0.07387	0.36	0.04	0.0008	0.03	0.20	0.36	1.00	1116	760	2.41	1.64
79.00	4	0.083035	0.33	0.04	0.0007	0.02	0.20	0.41	1.00	1090	721	3.12	2.06
80.00	5	0.094555	0.30	0.04	0.0007	0.01	0.18	0.47	1.00	1020	655	3.84	2.46

Appendix 2. The calculated probabilities of survival and mortality following the postoperative complications are illustrated for the routine fluid for five years. Each arrow represents a pathway and the pathways are characterized with probabilities. Each pathway is associated with costs and quality of life weights. The costs and quality of life weights of each pathway are weighted by the corresponding pathway probabilities. The sum of these weighted costs yields the expected cost during the first year and the weighted quality of life index multiplied by one year yields the expected quality adjusted life year (QALY). The cycles are repeated five times and the aggregated costs and QALYs are calculated. The same calculation is done for the GDHT.
